# The Antibacterial Efficacy and Mechanism of Plasma-Activated Water Against *Salmonella* Enteritidis (ATCC 13076) on Shell Eggs

**DOI:** 10.3390/foods9101491

**Published:** 2020-10-19

**Authors:** Chia-Min Lin, Chun-Ping Hsiao, Hong-Siou Lin, Jian Sin Liou, Chang-Wei Hsieh, Jong-Shinn Wu, Chih-Yao Hou

**Affiliations:** 1 Department of Seafood Science, National Kaohsiung University of Science and Technology, Kaohsiung 811, Taiwan; cmlin@nkust.edu.tw (C.-M.L.); darksevens728@gmail.com (H.-S.L.); 2 Department of Mechanical Engineering, National Chiao Tung University, Hsinchu 300, Taiwan; pandarugby@nctu.edu.tw (C.-P.H.); rinknew@gmail.com (J.S.L.); 3 Department of Food Science and Biotechnology, National Chung Hsing University, Taichung 402, Taiwan; welson@nchu.edu.tw

**Keywords:** plasma-activated water (PAW), *Salmonella*, egg

## Abstract

Eggs are one of the most commonly consumed food items. Currently, chlorine washing is the most common method used to sanitize shell eggs. However, chlorine could react with organic matters to form a potential carcinogen, trihalomethanes, which can have a negative impact on human health. Plasma-activated water (PAW) has been demonstrated to inactivate microorganisms effectively without compromising the sensory qualities of shell eggs. For this study, various amounts (250, 500, 750, or 1000 mL) of PAW were generated by using one or two plasma jet(s) at 60 watts for 20 min with an air flow rate at 6 or 10 standard liters per minute (slm). After being inoculated with 7.0 log CFU *Salmonella* Enteritidis, one shell egg was placed into PAW for 30, 60, or 90 s with 1 or 2 acting plasma jet(s). When 2 plasma jets were used in a large amount of water (1000 mL), populations of *S*. Enteritidis were reduced from 7.92 log CFU/egg to 2.84 CFU/egg after 60 s of treatment. In addition, concentrations of ozone, hydrogen peroxide, nitrate, and nitrite in the PAW were correlated with the levels of antibacterial efficacy. The highest concentrations of ozone (1.22 ppm) and nitrate (55.5 ppm) were obtained with a larger water amount and lower air flow rate. High oxidation reduction potential (ORP) and low pH values were obtained with longer activation time, more plasma jet, and a lower air flow rate. Electron paramagnetic resonance (EPR) analyses demonstrated that reactive oxygen species (ROS) were generated in the PAW. The observation under the scanning electron microscope (SEM) revealed that bacterial cells were swollen, or even erupted after treatment with PAW. These results indicate that the bacterial cells lost control of cell permeability after the PAW treatment. This study shows that PAW is effective against S. Enteritidis on shell eggs in a large amount of water. Ozone, nitrate, and ROS could be the main causes for the inactivation of bacterial cells.

## 1. Introduction

Eggs are a popular food choice in daily life and an excellent source of nutrients [1,2]. The major pathogenic microorganism associated with eggs is *Salmonella* spp. Salmonellosis associated with eggs is frequently reported in many countries [3]. In Taiwan, 77% of confirmed *Salmonella* cases were associated with eggs (TCDC, 2011). Among more than 2000 serovars, *S. enterica* serovar Enteritidis (S. Enteritidis) is the most frequently occurring serovar in eggs and egg products [4,5,6]. Thus, inactivating *Salmonella* on egg surfaces is a necessary process. Currently, the most common practice used to disinfect and clean the surface of shell eggs is washing with 100–200 ppm chlorinated water, alkaline detergent (such as quaternary ammonium salts), hot water, or steaming at 5–10 °C above the egg’s surface temperature. Chlorinated water is the most commonly used method, because of its relatively low cost and high efficacy. However, chlorine is not stable in high temperatures, and easily reacts with organic substances to form a potential carcinogen, trihalomethane [7]. In addition, modern consumers perceive chemical food treatments negatively. Thus, a novel technique to disinfect and clean shell eggs without chemical residue is strongly needed.

Non-thermal plasma is a novel physical method to disinfect microorganisms on foods without compromising sensory characteristics. Plasma ionizes the gas to generate charged particles, electric fields, ultraviolet (UV) photons, reactive nitrogen-oxygen (RNS), and reactive oxygen species (ROS), which are able to inactivate microorganisms [8]. Applying the plasma jet inside water, the charged particles, ROS, and RNS generated from gas ionization describe those which were previously easily dissolved in water. In addition, plasma also ionized the water to produce charged particles and ROS. These substances and the reaction products of these substances were effective antimicrobial agents. The substances are relatively non-stable and degrade rapidly, thus leaving no chemical residue [9,10]. This type of water is called plasma-activated water (PAW), which has been reported to inactivate foodborne pathogens on food-contact surfaces and food items without a negative impact to the environment and human health [8]. Several examples of using PAW to inactivate microorganisms on food have been reported, which include mushroom [11], strawberry [12], bean sprout [13], Korean rice cake [14], egg [15], and cabbage [16].

In past studies, several substances generated by the reactions of RNS and ROS in PAW were suggested to be the key agents in inactivating bacteria [17]. These agents included hydrogen peroxide (H_2_O_2_), singlet oxygen (O), nitrite (NO_2_^−^), and nitrate (NO_3_^−^). However, these potential antibacterial agents were not measured in the previous examples of using PAW on food items. Additionally, the amount of PAW used was relatively small (100–200 mL). Increasing the amount of PAW is a critical step towards industrial applications. Therefore, the objectives of this research were to evaluate the efficacy of larger amounts of PAW on shell eggs and investigate the potential antibacterial mechanism of PAW. To achieve these goals, different quantities of plasma generators and air flow rates were used in various amounts of water to obtain the optimal condition to inactivate *S*. Enteritidis on eggs. The concentrations of hydrogen peroxide (H_2_O_2_), ozone (O_3_), nitrite (NO_2_^−^), and nitrate (NO_3_^−^) in PAW was determined. The spectrum of plasma and the existence of singlet oxygen (O) were measured. Lastly, damage of the PAW-treated bacterial cells was observed under a scanning electron microscope.

## 2. Material and Methods

### 2.1. Preparation of Bacterial Suspension

The most commonly occurring pathogen associated with eggs, *Salmonella enterica* subsp. *enterica* serovar Enteritidis (ATCC 13076, Bioresource Collection and Research Centre, Taiwan), was used. This bacterium was maintained on tryptic soy agar (TSA) and confirmed by the selective media, xylose lysine deoxycholate agar (XLD). Fresh working culture was prepared by inoculating in tryptic soy broth (TSB), then incubated at 37 °C for 18–20 h consecutively, twice. Bacterial population density was maintained at a 1.0–1.5 optical density at 600 nm (OD_600_), which was approximately 9 log CFU/mL. The media used in this study were all purchased from Difco Laboratories (Detroit, MI, USA).

### 2.2. Preparation of Plasma-Activated Water (PAW)

**Figure 1:**
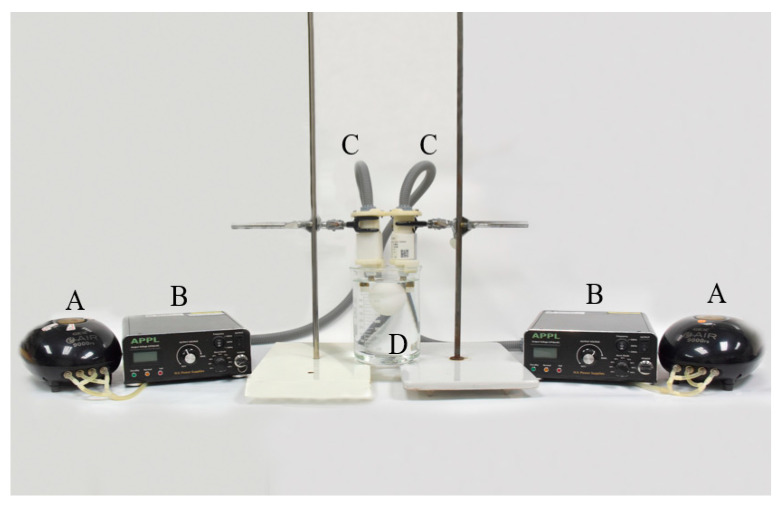
The devices of the air plasma-activated water system, including (**A**) air pumps, (**B**) high-voltage power supply, (**C**) plasma jet generators, and (**D**) the beaker containing one egg.

The device of the non-thermal atmospheric plasma generator was assembled by the Aerothermal and Plasma Physics Lab. (APPL), Department of Mechanical Engineering, National Chiao Tung University (Hsingchu, Taiwan). The major components of the system included a high-voltage power supply, an air pump, and an atmospheric pressure plasma jet (APPJ) (patent, US10,121,638B1) generator. During the study, single or duplicate system(s) were used to evaluate the effect of plasma jet quantities. This system was specifically designed to operate an electrode beneath the water surface to generate PAW. Atmospheric air was pumped into the electrode by the air pump, which also controlled the air flow rate. As per the previous study [15], reverse osmotic (RO) water was used as the water source. The voltage, frequency, pre-activation time, and power were set at 3.0 kV, 16 kHz, 20 min, and 60 Watts, respectively. The duplicate system used in this study is shown in Figure 1.

### 2.3. Inactivation of Salmonella *spp.* on Eggs by PAW

Eggs were obtained from a commercial egg farm with its own brand name. All testing eggs were unwashed and laid less than 2 days before testing. Only the eggs without surface debris, such as feces or other litter, were used. The chosen eggs were thoroughly washed by sterilized water, wiped by sterile paper, and then air-dried in a laminar hood. The washed egg was placed into a sampling bag with 100 mL phosphate buffer saline (PBS, pH 7.2). After being completely hand-rubbed for 2 min, decimally serial dilution and spreading on plate count agar (PCA) were used to check the presence of bacteria. Absence of *Salmonella* was confirmed based on the official method of the US Food and Drug Administration [18], in which the washed egg was placed into a sampling bag containing 225 mL lactose broth, then incubated at 37°C for 24 h. Tetrathionate (TT) broth and Rappaport-Vassiliadis (RV) broth were used as selective enrichment broth following the incubation of lactose broth. Selective agar media were xylose lysine deoxycholate (XLD), Hektoen enteric (HE), and bismuth sulfite (BS), which were used after the incubation of the selective enrichment broth. Before testing, S. Enteritidis (SE) culture was centrifuged at 5000× *g* for 5 min at 4 °C and resuspended in new TSB to achieve a population at 2–3 × 10^9^ CFU/mL. One hundred μL bacterial suspension was placed onto the surface of each egg in 33–35 drops, then air dried for 20–30 min. The inoculated eggs of the treatment group were transferred into a beaker containing pre-activated PAW. Different combinations of the operating parameters, such as air flow rate, water amount, treatment time, and plasma generator quantity, were tested to obtain the optimal conditions (Table 1). The treated egg was transferred into a sterile sampling bag with 100 mL of PBS, then gently rubbed by hand for 2 min. After being decimally serial-diluted, 0.1 mL of the PBS was spread onto PCA. The plates were incubated at 37 °C for 18–24 h, and triplicate plates were used for each dilution. Two to three colonies on a PCA plate which were randomly re-streaked onto XLD agar to confirm the recovered bacteria were *Salmonella*. Inoculated eggs washed with the same amount of sterile RO water with the same time were used as the water washing control. Inoculated eggs without washing were used as the negative control [15].

### 2.4. Measurement of pH, Oxidative-Reductive Potential (ORP), Hydrogen Peroxide (H_2_O_2_), Ozone (O_3_), Nitrite (NO^2−^), and Nitrate (NO^3−^) of PAW

The values of pH, oxidative-reductive potential (ORP), conductivity, hydrogen peroxide (H_2_O_2_), ozone (O_3_), nitrite (NO^2−^), and nitrate (NO^3−^) were determined to investigate the physio-chemical characteristics of PAW.

#### 2.4.1. Measurement of pH and Oxidative-Reductive Potential (ORP)

The values of pH and oxidative-reductive potential (ORP) were measured by a pH/ORP meter (PC-200, Cole-Parmer, Vernon Hills, IL, USA) connected with a pH or ORP probe (serial 100 probe, Cole-Parmer).

#### 2.4.2. Measurements of Hydrogen Peroxide (H_2_O_2_)

The concentration of H_2_O_2_ at each treatment time, 30, 60, and 90 s was measured according to the method of the Department of Health and Welfare, Taipei City, Taiwan (2013). Five mL of PAW was collected immediately after treatment and mixed with 0.1% *o*-phenylenediamine (OPD) in citric acid buffer (pH 5.0). After reacting for 10 min, 1 mL 10 N sulfuric acid was added, and absorbance at 490 nm was recorded. The H_2_O_2_ concentrations were determined by comparing with the standard curve ranging from 0 to 6.0 mg/L. The absorbance at 490 nm was adjusted by using sterile RO water as the testing sample.

#### 2.4.3. Measurement of Ozone (O_3_)

Ozone concentrations at each treatment time, 30, 60, and 90 s, were determined by Ozone AccuVac^®^ Ampuls (0–1.5 mg/L) in a handheld colorimeter (DR900, HACH, Loveland, CO, USA). Following manufactural instruction, 40 mL PAW was sampled immediately after treatment and the ampule containing the reagents was placed into the tilted PAW sample. After the ampule was filled with PAW, it was sealed by a rubber stopper and shaken until the reagents totally dissolved. After reacting for 1 min, the concentrations were measured in the colorimeter. Sterile RO water was used as the blank.

#### 2.4.4. Measurement of Nitrite (NO^2−^), and Nitrate (NO^3−^)

The concentrations of nitrate and nitrite at each treatment time, 30, 60, and 90 s were measured by Nitrite Reagent Powder Pillows (2–250 mg/L NO^2−^, and 0.3–30 mg/L NO^3−^) in a handheld colorimeter (DR900, HACH, Loveland, CO, USA). As per the manufactural instructions, 10 mL of PAW was sampled and mixed with the powder pillows. After reacting for 5 min, the concentrations of NO^2−^ and NO^3−^ were determined in the colorimeter. Sterile RO water was used as the blank.

### 2.5. Measurement of the Optical Characteristics of Plasma by Optical Emission Spectroscopy (OES)

The optical characteristics of the plasma jet in the water was measured by an emission spectroscope (SP-2500, Acton Research, Acton, MA, USA). A plasma jet was placed beneath the water surface in a quartz tube. Plasma was discharged beneath the water. A fiber optics cable connected with the spectroscope was used to measure the optical signals with wavelengths between 200 to 850 nm.

### 2.6. Detection of Reactive Oxygen Species (ROS) by Electron Paramagnetic Resonance (EPR)

The generation of ROS in the PAW was determined by the reaction of superoxide, as well as superoxide-related formations of hydroxyl radicals within the spin trap of DMPO (5,5-dimethylpyrroline-N-oxide). Magnetic fields strength (G) of the sin trap was detected by an electron paramagnetic resonance (EPR) spectrometer (EMXplus-10/12/P/L system, Bruker Inc., Billerica, MA, USA). PAW was generated on site, and 1 L RO water was activated for 20 min using two plasma jets with an air flow rate at 10 slm. After 20 min activation, 1 mL PAW was collected at 30, 60, and 90 s with the plasma jets were still acting. After mixing with 20 μL of 1 M DMPO, the samples were immediately measured by EPR. RO water added with the same amount of DMPO was used as the control [19].

### 2.7. Observation Under Scanning Electron Microscope (SEM)

The inoculated eggs were treated with PAW for 60 or 90 s in 1 L PAW, as described previously, and unwashed inoculated eggs were used as the control. After treatment, eggshells were collected, and the inner membrane was removed. The shells were immersed in a phosphate buffer containing 2.5% glutaraldehyde at 4 °C for 2 h, then further washed three times with phosphate buffer and deionized water. The washed shells were soaked in gradually increasing concentrations of ethanol solutions (50%, 70%, 80%, 90%, and 95%). The dehydrated eggshell was further treated at −20 °C for 2 h, then −80 °C for 12 h. Finally, the shell samples were freeze-dried for 4 h, then stored in a desiccator before SEM observation. The samples were observed under a Quanta 200 Environmental Scanning Electron Microscope (Thermo Fisher Scientific, Waltham, MA, USA).

### 2.8. Statistical Analyses

Each treatment had triplicate samples, and all experiments were conducted at least twice. Data were analyzed by using one-way ANOVA and Duncan’s test was used for post hoc analysis. The significant differences between treatments were set at *p* < 0.05. All statistical analyses were conducted by using an IBM SPSS program (Version 22.0, St. Armonk, NY, USA).

## 3. Results

### 3.1. The Effects of Plasma Jet Quantity, Treatment Time, and Air Flow Rates

While one plasma jet was used, bactericidal activities increased with longer treatment time (*p <* 0.05) and reduced significantly when large water amounts were tested (*p <* 0.05). When compared with egg samples washed by sterile water under the same treatment time, 2.82–3.07 and 1.92–2.89 log CFU/egg reductions were obtained from the PAW at 250 and 500 mL, respectively. However, only 0.53–0.92 log and 0–0.08 CFU/egg reductions were obtained at 750 and 1000 mL (Table 2). Bactericidal activities increased greatly when two plasma jets were used. More than five log reductions were obtained when eggs were treated for 90 s in 750 and 1000 mL PAW (Table 2). Longer treatment time still resulted in greater reductions (*p <* 0.05). However, no significant difference was observed between different water volumes at the same treatment time (*p* > 0.05).

Since the PAW generated with two plasma jets was more effective and a large water volume was the objective, the effects of different air flow rates were only tested in the two plasma jet systems at the 1000 mL water amount. Significantly lower reduction (*p <* 0.05) was obtained from the samples using 10 slm rather than 6 slm air flow rate for all three treatment time-periods. When treatment time was extended from 60 s to 90 s, no significant decrease (*p <* 0.05) in bacterial populations were obtained at both air flow rates (Table 3).

### 3.2. The pH and Oxidative-Reductive Potential (ORP) Values of PAW

Within the PAW generated by one plasma jet, the values of pH decrease and ORP increase were reduced significantly (*p* < 0.05) when a larger water volume was used. However, when adding one more plasma jet, the pH and ORP values of the PAW were not significantly different (*p >* 0.05) between various water amounts (Table 4). However, when the PAW was generated with two plasma jets, significantly lower ORP values were obtained with the high air flow rate (10 slm) (Table 5).

### 3.3. The Concentrations of Ozone (O_3_), Hydrogen Peroxide (H_2_O_2_), Nitrate (NO_3_^−^), and Nitrile (NO^2−^) in PAW

Both the concentrations of O_3_ and H_2_O_2_ were lower at 10 slm compared to 6 slm, particularly H_2_O_2_, whose values were significantly lower (*p* < 0.05) at 10 slm. When the water amount increased, the variation of O_3_ concentrations was insignificant (*p* > 0.05). However, H_2_O_2_ concentrations reached the highest at 750 mL, but decreased markedly at 1000 mL (Table 6).

The NO_2_^−^ concentrations were higher than NO_3_^−^ in PAW (Table 7). Both NO_3_^−^ and NO_2_^−^ concentrations increased with longer treatment time. At different air flow rates, NO_3_^−^ concentrations increased at 10 slm, but NO_2_^−^ concentrations decreased at 10 slm. However, NO_3_^−^ concentrations decreased when the water volume increased, but NO_2_^−^ concentrations increased along with the water volume.

### 3.4. The Optical Characteristics of Plasma and the Reactive Oxygen Species (ROS) in PAW

**Figure 2:**
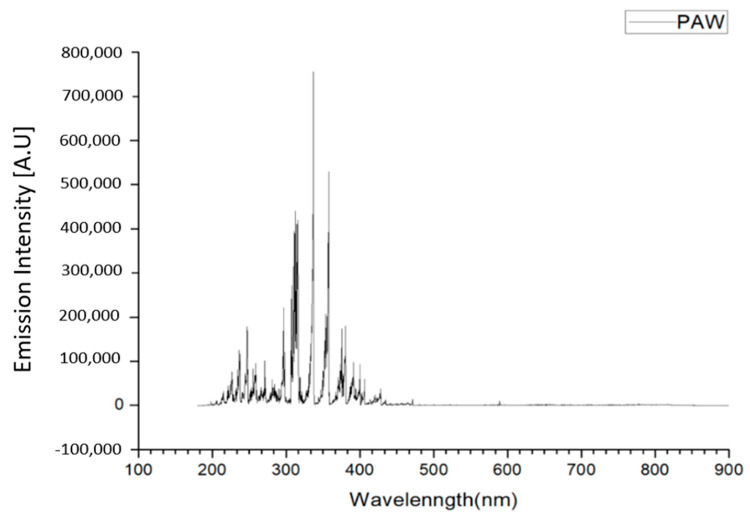
Space-integrated optical spectra of the plasma for water activation.

The spectrum of the plasma jet in the PAW was from 190 to 460 nm. Most of the spectrum was located in the wavelength range of ultraviolet light (UV) (91.25%). Further examination showed UVA (320 nm–400 nm) and UVB (275-320 nm) were the major parts (66.6%). The shorter wavelength UVC (100–275 nm) that possesses higher antibacterial ability was the minor part (25.19%) (Figure 2).

**Figure 3:**
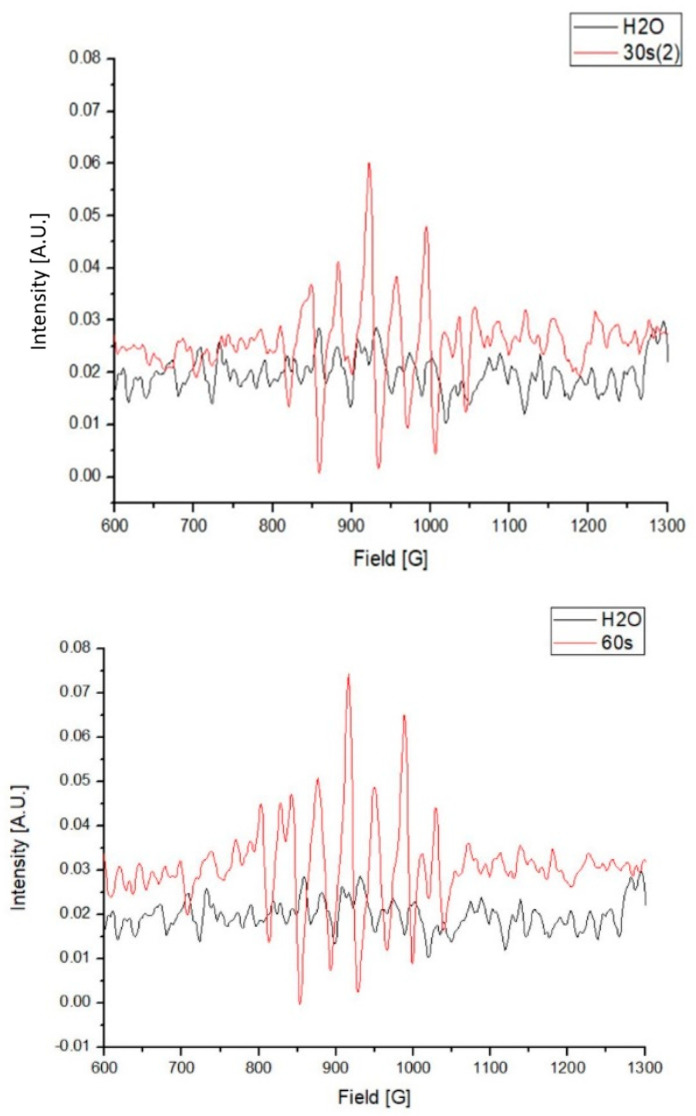

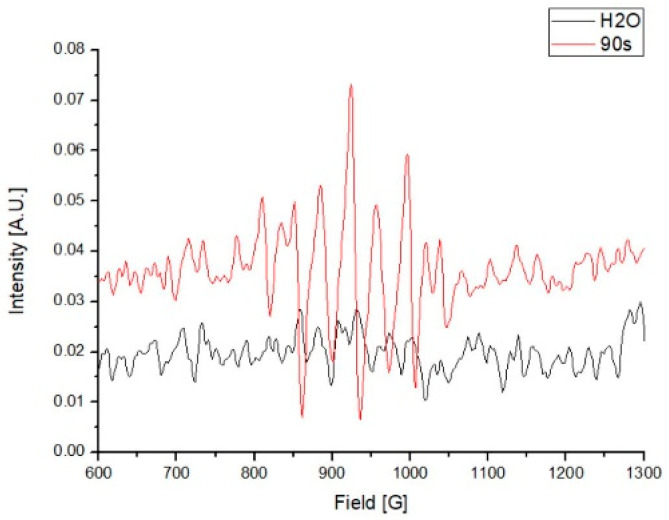
Electron paramagnetic resonance (EPR) of the plasma-activated water (PAW) in 1000 mL.

Compared with the water control, several higher absorbance peaks, from between 810 to 1040 G, were observed in the PAW. These results indicate the existence of unpaired electrons in the PAW, which were predominantly generated by the activation of plasma in the water (Figure 3).

### 3.5. The Observation of Scanning Electronic Microscope (SEM)

**Figure 4:**
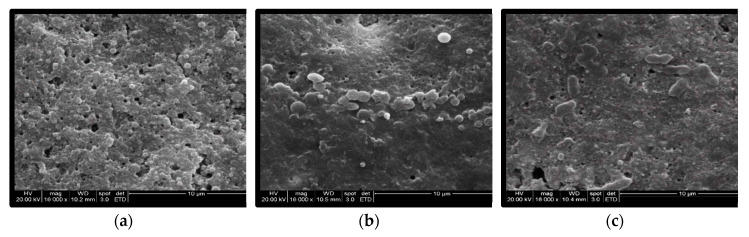
SEM observation of the inoculated eggshells (×16,000 magnification): (**a**) Unwashed, (**b**) PAW 60 s, (**c**) PAW 90 s.

After PAW treatment, most *Salmonella* cells on the egg surface were much larger than the cells on the untreated eggs. In addition, some bacterial cells were even ruptured (Figure 4).

## 4. Discussion

Increasing the water volume is an essential step for practical use of PAW. In this study, the water volume increased to 1 L which was larger than most studies using PAW to inactivate foodborne pathogens on foods [15,20,21,22,23]. Among the reports that used 1 L of water, Han et al. (2020) used two plasma jets in 1 L of distilled water for 20 min, then treated Korean rice cakes for 20 min [14]. Choi et al. (2019) used a device containing 12 pins of discharged plasma over the surface of 1 L of distilled water for 30, 60, and 120 min, then treated cabbages for 10 min [16]. Lastly, Liu et al. (2020) submerged 28 acting plasma jets in 1 L of distilled water for 10 min, then treated fresh-cut apples for 5 min [24]. In this study, two plasma jets were used to activate 1 L of PAW that was able to inactivate more than 3 log SE on egg surfaces after 60 s treatment. The number of plasma jets used was less, and the sample treatment time was also shorter than previous studies at the same water volume. Thus, the use of electrical energy in this study was more efficient.

Several PAW studies combined PAW treatment with mild heat (50–60 °C) to enhance antibacterial activity. In the study on cabbages [16], treatment with the PAW activated under 120 min (PAW-120) for 10 min was able to reduce 2.0, 2.2, 1.8, and 0.9 log for mesophilic bacteria, lactic acid bacteria, yeast and mold, and coliform, respectively. Subsequent treatment in distilled water at 60 °C for 5 min resulted in greater pathogen reduction. When the cabbage was inoculated with *L. monocytogenes* and *Staphylococcus aureus*, 3.4 and 3.7 log reduction was obtained, respectively, after the combined treatment of PAW-120 and 60 °C water. Xiang et al. (2020) applied 30 mL of APW at 50–55 °C for 30 min to treat grapes and obtained more than 5 log reduction of *Saccharomyces cerevisiae*. During this research, the PAW temperature raised naturally to 40–42 °C for all treatments. Thus, the treatment temperatures of this study were lower than the previous studies, in which 50–60 °C [16] and 50–55 °C [21] were used for treating cabbage and grapes, respectively. The PAW temperature was mainly affected by the type of plasma, number of plasma jets, and treatment time. Generally, the temperature was lower when using noble gas, such as argon, instead of regular air [9]. Longer treatment time and a higher number of plasma jets induced higher temperatures [15,17]. In this study, the temperature of smaller-volume PAW (250 mL) was slightly higher than large-volume PAW (1000 mL). However, the temperature difference was not significant, and all were within the range of 40–42 °C. Unlike many other food items, the temperature of the egg washing solution must be higher than the temperature of the egg surface to prevent the infiltration of surface bacteria into the egg [25]. Therefore, the PAW temperature used in this research was in the same temperature range used in commercial egg washing. The RO water used in the controls were also raised to the same temperature as the PAW. The RO control groups obtained a SE reduction of around 1 log when washed at 40–42 °C. In a preliminary study, the same amount of SE reduction was achieved when 25–28 °C RO water was used. Thus, the SE reduction obtained from PAW treatments was caused by the PAW, and not the elevated temperature at 40–42 °C.

The main mechanisms of PAW to inactivate microorganisms were reported to be the creation of reactive oxygen species (ROS), reactive nitrogen species (RNS), and UV light [26]. In this study, the existence of ROS was detected by EPR. The photo spectrum showed that the majority of the light generated by the plasma was within the range of UV light. It has been reported that the UV light generated from the acting plasma under water contributed to the bacterial inactivation [27]. The ROS generated during the PAW activation was also confirmed by previous studies [19]. Moreover, Wu et al. (2017) demonstrated that adding H_2_O_2_ simultaneously enhanced the bactericidal activity and the intensity of the magnetic field, which provided a direct connection between the existence of ROS and the bactericidal ability of PAW [19]. In our study, the EPR results also showed that higher intensity was obtained from 60 and 90 s of treatment time compared to 30 s, which corresponded with the antibacterial results. The majority of ROS are hydrogen peroxide (H_2_O_2_) and oxygen molecules containing unpaired electrons, such as atomic oxygen (O), ozone (O_3_), superoxide (O_2_^−^), hydroxyl radicals (OH^●^), and singlet oxygen (^1^O^2^). Nitrate and nitrile were the major products of the reaction between RNS and these active oxygen molecules [28]. The production of these substances result in the increase of ORP and the decrease of pH [28,29]. Thus, ORP and pH values were used in many studies [17,19,28,30] to monitor the antibacterial activity of PAW, including our previous study [15]. In this study, the increase of ORP values and decrease of pH values became less as more water was added in PAW activated by one plasma jet. In contrast, the ORP and pH values were not significantly different between various water volumes in the PAW activated by two plasma jets. These results corresponded with the efficacy of SE reduction. When the PAW was activated with two jets in 1 L of water at 6 or 10 slm, the higher air flow rate not only diminished the ORP values, but also the O_3_, H_2_O_2_, and NO_2_^−^ concentrations, particularly the H_2_O_2_ concentrations. Our results also showed the reductions of SE populations were less when PAW was activated at 10 slm than 6 slm. Thus, the concentrations of O_3_, H_2_O_2_, and NO_2_^−^ were related with the bactericidal activity of PAW, which was also shown in previous studies [17,19,31,32]. Though NO_3_^−^ concentrations slightly increased at 10 slm, its concentrations were far less than the NO_2_^−^ concentrations.

SEM observation also revealed that the inoculated SE cells inflated, or even erupted. The possible reason for this could be that the cell membrane lost the ability to maintain osmotic balance, which resulted in the extracellular water infiltrating into the cell continuously. Several reports have described that the impairment of the cell membrane was the major antibacterial mechanism of PAW and resulted in higher permeability, which caused the leakage of intracellular substances [13,33]. However, this study was the first one to demonstrate the enlargement of the treated bacterial cells after PAW treatment. Since the main components of the cell membrane are phospholipids, the UV light, ROS, and the acidic substances generated by PAW were all able to injure phospholipids and cause the loss of the cell membrane’s permeability control [13,33]. Besides the impairment of the cell membrane, damage to bacterial DNA and their metabolism were also reported [9,32,34].

The results of this study demonstrate that a large-scale PAW generation system is achievable, and provide a foundation for future practical use, in which a larger and faster production of PAW is essential. In addition, the antibacterial efficacy of PAW obtained in this study was greater than the study using sodium chlorine and chlorine dioxide [35]. Furthermore, it offered a direct correlation between the PAW products described previously and its antibacterial capacity, which also presented a clearer road map to understanding the antibacterial mechanisms of PAW.

## 5. Conclusions

A patented air plasma jet system was used to generate 1 L of PAW under various conditions to inactivate SE on egg surfaces. The results showed that using two plasma jets at 6 slm was the optimal condition. The values of ORP and pH were related with the antibacterial efficacy. The products generated during PAW activation, such as UV light, unpaired oxygen electrons, O_3_, H_2_O_2_, nitrile, and nitrate were detected. Furthermore, the relationship between the quantitative amount of these products and the antibacterial efficacy of PAW was demonstrated. The observation under SEM revealed SE cells were inflated, which was mainly caused by the loss permeability of the cytomembrane. The results of this study indicated that air-generated PAW could be a practical option to clean and disinfect shell eggs. More evidence regarding the connection between the PAW products and PAW’s antibacterial activity was also provided.

## Figures and Tables

**Table 1:** The operating parameters of plasma-activated water (PAW) to inactivate *S*. Enteritidis on shell eggs.

Parameters	Variance	Unit
Air flow rate	6, 10	standard liter per min (slm)
Water amount	250, 500, 750, 1000	mL
Treatment time	30, 60, 90	sec
Plasma jet number	1, 2	

**Table 2:** The populations of *Salmonella* Enteritidis (log CFU/egg) on eggs treated by the plasma-activated water (PAW) generated with one or two plasma jets at 6 slm air flow with different treatment times and volumes.

Treatments/Volume	250 mL	500 mL	750 mL	1000 mL
Unwashed	7.22 ± 1.04 ^aA^	7.37 ± 0.60 ^aA^	8.06 ± 0.06 ^aA^	7.92 ± 0.25 ^aA^
Sterile water-30s	6.85 ± 0.23 ^bA^	6.69 ± 0.42 ^bA^	7.12 ± 0.21 ^bA^	7.06 ± 0.01 ^bA^
Sterile water-60s	6.66 ± 0.06 ^bA^	6.60 ± 0.06 ^bA^	6.63 ± 0.16 ^bA^	6.54 ± 0.34 ^bcA^
Sterile water-90s	6.24 ± 0.27 ^bA^	6.49 ± 0.27 ^bA^	6.43 ± 0.27 ^bA^	6.36 ± 0.16 ^bcA^
One plasma jet				
PAW-30s	3.78 ± 0.64 ^cdA^	4.77 ± 0.92 ^cB^	6.49 ± 0.13 ^bC^	6.98 ± 0.12 ^bcC^
PAW-60s	4.27 ± 0.63 ^cA^	4.26 ± 0.67 ^cdA^	6.10 ± 0.11 ^bcB^	6.59 ± 0.27 ^bcB^
PAW-90s	3.42 ± 0.37 ^cdA^	3.60 ± 0.65 ^dA^	5.51 ± 0.27 ^cB^	6.28 ± 0.19 ^cC^
Two plasma jet				
PAW-30s	4.55 ± 0.60 ^cA^	4.33 ± 0.15 ^cdA^	4.36 ± 0.41 ^dA^	4.67 ± 0.32 ^dA^
PAW-60s	3.48 ± 0.66 ^cdA^	3.87 ± 0.39 ^dA^	3.73 ± 0.48 ^dfA^	2.84 ± 1.34 ^eA^
PAW-90s	3.16 ± 0.10 ^dA^	3.38 ± 0.32 ^dA^	2.69 ± 0.37 ^fA^	2.78 ± 0.56 ^eA^

Data are presented as average ± STD. Average in the same column with different lower-case letters are significantly different (*p* < 0.05). Average in the same row with different upper-case letters are significantly different (*p* < 0.05).

**Table 3:** The populations of *Salmonella* Enteritidis (log CFU/egg) on eggs treated in 1000 mL plasma-activated water (PAW) generated with two jets at 6 slm or 10 slm.

Treatments/Flow Rate	6 slm	10 slm
Unwashed	7.24 ± 0.23 ^Aa^	7.71 ± 0.11 ^Aa^
Sterile water-30s	7.06 ± 0.01 ^Aa^	6.69 ± 0.17 ^Ab^
Sterile water-60s	6.54 ± 0.34 ^Aa^	6.46 ± 0.06 ^Ab^
Sterile water-90s	6.23 ± 0.16 ^Aa^	6.34 ± 0.13 ^Ab^
PAW-30s	4.67 ± 0.32 ^Ab^	5.20 ± 0.16 ^Bc^
PAW-60s	2.84 ± 1.34 ^Ac^	4.53 ± 0.17 ^Bd^
PAW-90s	2.78 ± 0.56 ^Ac^	3.83 ± 0.07 ^Bd^

Data are presented as average ± STD. Average in the same column with different lower-case letters are significantly different (*p* < 0.05). Average in the same row with different upper-case letters are significantly different (*p* < 0.05).

**Table 4:** The pH and oxidative-reduction potential (ORP) values of the plasma-activated water (PAW) generated with one plasma jet and two plasma jets at various treatment times and water volumes.

Treatments/Volume		250 mL	500 mL	750 mL	1000 mL
RO water	pH	6.23 ± 0.20 ^aA^	6.27 ± 0.28 ^aA^	6.25 ± 0.21 ^aA^	6.13 ± 0.13 ^aA^
	ORP	200.9 ± 3.5 ^dA^	249.4 ± 6.1 ^dA^	254.6 ± 28.7 ^dA^	220.4 ± 13.5 ^dA^
One plasma jet					
PAW-30s	pH	3.36 ± 0.07 ^bA^	3.43 ± 0.17 ^bA^	3.53 ± 0.15 ^bA^	3.95 ± 0.11 ^bB^
	ORP	492.5 ± 14.1 ^cA^	499.8 ± 7.1 ^cA^	476.2 ± 8.6 ^cB^	467.8 ± 7.5 ^cB^
PAW-60s	pH	3.29 ± 0.10 ^bA^	3.46 ± 0.16 ^bA^	4.01 ± 0.04 ^bA^	3.87 ± 0.03 ^bA^
	ORP	503.2 ± 18.6 ^bA^	513.9 ± 11.2 ^bA^	505.5 ± 7.2 ^bA^	491.1 ± 11.3 ^bA^
PAW-90s	pH	3.41 ± 0.10 ^bA^	3.55 ± 0.14 ^bA^	3.71 ± 0.08 ^bB^	3.81 ± 0.06 ^bB^
	ORP	531.6 ± 3.4 ^aA^	536.7 ± 10.3 ^aA^	496.8 ± 17.3 ^aB^	509.6 ± 2.2 ^aB^
Two plasma jet					
PAW-30s	pH	3.20 ± 0.08 ^bA^	3.12 ± 0.02 ^bA^	3.24 ± 0.08 ^bA^	3.29 ± 0.02 ^bA^
	ORP	540.5 ± 0.4 ^aA^	543.20 ± 0.1 ^aA^	530.8 ± 12.7 ^aA^	505.95 ± 1.2 ^aB^
PAW-60s	pH	3.10 ± 0.02 ^bA^	3.17 ± 0.09 ^bA^	3.16 ± 0.03 ^bA^	3.20 ± 0.02 ^bA^
	ORP	510.2 ± 8.1 ^bA^	534.0 ± 1.4 ^bA^	523.6 ± 7.6 ^bA^	515.7 ± 2.1 ^bA^
PAW-90s	pH	3.06 ± 0.01 ^cA^	3.08 ± 0.05 ^cA^	3.12 ± 0.06 ^cA^	3.25 ± 0.08 ^cA^
	ORP	529.5 ± 6.2 ^bA^	533.7 ± 10.7 ^bA^	520.7 ± 23.3 ^bA^	527.1 ± 9.7 ^bA^

Data are presented as average ± STD. Average in the same column for the same testing with different lower-case letters are significantly different (*p* < 0.05). Average in the same row with different upper-case letters are significantly different (*p* < 0.05).

**Table 5:** The values of pH and oxidative-reduction potential (ORP) of 1000 mL PAW generated by two plasma jets at different treatment times and flow rates.

Treatments/Flow Rate		6 slm	10 slm
RO water	pH	7.91 ± 0.12 ^aA^	8.05 ± 0.23 ^aA^
	ORP	239.6 ± 18.10 ^cA^	210.1 ± 12.5 ^bA^
PAW-30s	pH	3.29 ± 0.02 ^bA^	3.31 ± 0.10 ^bA^
	ORP	505.95 ± 1.2 ^aA^	465.2 ± 4.1 ^aB^
PAW-60s	pH	3.20 ± 0.02 ^bA^	3.40 ± 0.02 ^bA^
	ORP	515.70 ± 2.12 ^bA^	488.8 ± 7.4 ^aB^
PAW-90s	pH	3.25 ± 0.08 ^cA^	3.32 ± 0.03 ^bA^
	ORP	527 ± 9.76 ^bA^	501.2 ± 24.6 ^aB^

Data are presented as average ± STD. Average in the same column for the same testing with different lower-case letters are significantly different (*p* < 0.05). Average in the same row with different upper-case letters are significantly different (*p* < 0.05).

**Table 6:** The concentrations of ozone (O_3_) and hydrogen peroxide (H_2_O_2_) of the PAW generated by two plasma jets at 6 or 10 slm with different treatment times and water volumes.

slm	Time/Volume	250 mL	500 mL	750 mL	1000 mL
O_3_					
6	30 s	1.03 ± 0.05 ^bA^	0.92 ± 0.03 ^bA^	0.84 ± 0.02 ^aA^	0.90 ± 0.06 ^aA^
	60 s	1.13 ± 0.12 ^aA^	1.18 ± 0.05 ^aA^	0.93 ± 0.09 ^aA^	0.96 ± 0.04 ^aA^
	90 s	1.12 ± 0.02 ^aA^	1.22 ± 0.02 ^aA^	0.96 ± 0.52 ^aA^	1.16 ± 0.18 ^aA^
10	30 s	0.91 ± 0.04 ^aA^	0.94 ± 0.01 ^aA^	0.87 ± 0.01 ^aA^	0.75 ± 0.06 ^bA^
	60 s	0.99 ± 0.05 ^aA^	0.90 ± 0.08 ^aA^	0.93 ± 0.04 ^aA^	0.60 ± 0.02 ^bA^
	90 s	0.97 ± 0.02 ^aA^	1.02 ± 0.02 ^aA^	0.91 ± 0.60 ^aA^	0.71 ± 0.54 ^bA^
H_2_O_2_					
6	30 s	11.78 ± 0.73 ^a^	17.84 ± 0.44 ^a^	21.16 ± 0.70 ^a^	18.03 ± 0.71 ^a^
	60 s	14.95 ± 1.02 ^a^	17.52 ± 0.70 ^a^	20.83 ± 0.68 ^a^	18.27 ± 0.75 ^a^
	90 s	11.42 ± 0.77 ^a^	16.73 ± 0.25 ^a^	20.22 ± 0.86 ^a^	17.49 ± 0.39 ^a^
10	30 s	2.96 ± 0.03 ^b^	2.91 ± 0.06 ^b^	2.89 ± 0.11 ^b^	1.88 ± 0.15 ^b^
	60 s	2.76 ± 0.01 ^b^	2.89 ± 0.08 ^b^	3.06 ± 0.07 ^b^	1.91 ± 0.49 ^b^
	90 s	2.72 ± 0.04 ^b^	2.78 ± 0.07 ^b^	2.75 ± 0.17 ^b^	1.65 ± 0.14 ^b^

Data are presented as average ± STD. Average in the same column for the same testing with different lower-case letters are significantly different (*p* < 0.05). Average in the same row with different upper-case letters are significantly different (*p* < 0.05).

**Table 7:** The concentrations of nitrate (NO_3_^−^) and nitrite (NO_2_^−^) of the PAW generated by two plasma jets at 6 or 10 slm with different treatment times and water volumes.

slm	Time/Volume	250 mL	500 mL	750 mL	1000 mL
NO_3_^−^					
6	30 s	5.20 ± 0.60 ^bA^	4.56 ± 0.20 ^bB^	4.60 ± 1.25 ^aB^	5.01 ± 0.17 ^aAB^
	60 s	4.96 ± 0.68 ^bA^	4.53 ± 0.37 ^aA^	4.46 ± 0.21 ^cA^	4.71 ± 0.11 ^aA^
	90 s	7.26 ± 0.61 ^aA^	6.70 ± 0.05 ^bA^	6.06 ± 0.20 ^bB^	4.70 ± 0.25 ^aC^
10	30 s	5.46 ± 0.40 ^bB^	5.70 ± 0.10 ^aB^	6.53 ± 0.05 ^bA^	4.70 ± 0.30 ^bC^
	60 s	5.40 ± 0.36 ^bA^	5.90 ± 0.34 ^aA^	5.33 ± 0.15 ^cA^	5.56 ± 0.15 ^aA^
	90 s	9.66 ± 0.72 ^aA^	5.63 ± 0.10 ^aB^	8.10 ± 0.23 ^aA^	5.93 ± 0.26 ^aB^
NO_2_^−^					
6	30 s	40.5 ± 0.70 ^aB^	43.5 ± 0.90 ^aB^	49.5 ± 0.40 ^aA^	55.5 ± 0.37 ^aA^
	60 s	42.5 ± 0.60 ^aB^	44.5 ± 0.50 ^aB^	50.5 ± 1.53 ^aA^	53.0 ± 0.80 ^aA^
	90 s	40.0 ± 0.24 ^aB^	43.5 ± 1.06 ^aB^	46.5 ± 1.76 ^aB^	54.5 ± 0.35 ^aA^
10	30 s	34.1 ± 1.41 ^bB^	31.5 ± 0.32 ^bB^	44.5 ± 0.40 ^bA^	47.5 ± 0.30 ^bA^
	60 s	35.5 ± 0.60 ^bB^	31.0 ± 1.41 ^bB^	44.0 ± 1.41 ^bA^	46.5 ± 0.70 ^bA^
	90 s	35.4 ± 0.10 ^bB^	36.0 ± 0.35 ^bB^	45.0 ± 1.10 ^bA^	48.0 ± 0.51 ^bA^

Data are presented as average ± STD. Average in the same column for the same testing with different lower-case letters are significantly different (*p* < 0.05). Average in the same row with different upper-case letters are significantly different (*p* < 0.05).
